# Editorial: An Omics Perspective on Fungal Infection: Toward Next-Generation Diagnosis and Therapy

**DOI:** 10.3389/fmicb.2017.00085

**Published:** 2017-01-26

**Authors:** Agostinho Carvalho, Gustavo H. Goldman

**Affiliations:** ^1^Life and Health Sciences Research Institute (ICVS), School of Medicine, University of MinhoBraga, Portugal; ^2^ICVS/3B's - PT Government Associate LaboratoryBraga/Guimarães, Portugal; ^3^Faculdade de Ciências Farmacêuticas de Ribeirão Preto, Universidade de São PauloSão Paulo, Brazil

**Keywords:** omics techniques, fungal infection, personalized healthcare, antifungal therapeutics, biomarker discovery

Fungal infections are estimated to occur in over a billion people each year, and evidence suggests the rate is increasing. Vaccines are unavailable, and despite progress in diagnosis and therapy, the management of fungal infections is a challenging endeavor associated with unacceptable mortality rates, particularly in immunocompromised hosts (Kontoyiannis et al., 2010; Pagano et al., 2010). Importantly, the risk of infection and its clinical outcome vary significantly even among patients with similar predisposing conditions (Carvalho et al., 2010). Concerns over excessive prescription of antifungals and the remarkable burden conveyed to healthcare systems have encouraged efforts to decipher the molecular and cellular causes underlying variable susceptibility to infection (Cunha and Carvalho, 2012).

The combination of omics technologies and advanced computational methods, together with the use of both established and alternative *in vitro* and *in vivo* models of infection (Brunke et al., 2015), provides comprehensive views of the architecture and dynamics of host-fungus interaction networks at a level of complexity previously unanticipated. As a result of our deepened understanding of the biological, biochemical and biophysical molecular processes regulating the host-fungus interaction, several targets with potential usefulness in personalized medical interventions have been proposed (Oliveira-Coelho et al., 2015).

The present Research Topic brings together 10 articles covering multiple aspects of the host-fungus interaction with emphasis on the application of omics-based technologies to project novel or improve current diagnostic and therapeutic approaches. Smeekens et al. point out the advent of omics platforms and the development of systems biology tools to study antifungal immunity (Smeekens et al.). Much research has been performed on host genetics and fungal infection (Cunha et al., 2013; Smeekens et al., 2013b), but only recently have these data been integrated into functional genomics approaches driving unbiased identification and quantification of targets controlling susceptibility to infection (Smeekens et al., 2013a; Kumar et al., 2014). With the increasing number and quality of data repositories, the generation of multi-scale host-fungus interaction models through systems biology is expected to support personalized medicine interventions (Dix et al., 2016).

A substantial proportion of genetic markers associated with the risk of fungal infection are within immune-related genes (Lupianez et al., 2015), often implicated in the nuclear factor (NF)-κB signaling pathways. Through a systematic evaluation of single nucleotide polymorphisms (SNPs) in NF-κB pathways, Lupiañez et al. exclude a clinically relevant impact of SNPs in these genes to the risk of invasive aspergillosis (IA) (Lupiañez et al.), thus supporting the concept that innate immune receptors, rather than molecules involved in downstream signaling, are major repositories of genetic variability regulating antifungal immune function (Bochud et al., 2008; Carvalho et al., 2008, 2012b; Cunha et al., 2010, 2014, 2015; Wojtowicz et al., 2015). As host damage perception is fundamental for resolution of infection (Cunha et al., 2012), SNPs underlying the hyperactivation of the S100 calcium-binding protein B (S100B)/receptor for advanced glycation end products danger signaling pathway have also been put forward as intrinsic factors influencing the risk for IA (Cunha et al., 2011). Accordingly, Dix et al. describe an enrichment of gene expression profiles of patients suffering from IA in the S100B transcript, therefore highlighting its potential as a valuable prognosis biomarker (Dix et al.).

The use of transcriptomics and epigenomics has also contributed to the identification and characterization of dynamic cellular processes with unparalleled resolution. An emerging view is that immune cells are able to adapt their metabolic programs to meet specialized defense needs through the precise and concerted action of epigenetic mechanisms and metabolic pathways (Cheng et al., 2014; Saeed et al., 2014). Hellwig et al. provide evidence that the effector functions of natural killer T cells in response to *Candida albicans* are also critically dependent on metabolic plasticity (Hellwig et al.). This finding is in line with the metabolic reprogramming and epigenetic imprinting occurring in monocytes in response to β-glucan (Netea et al., 2016). Understanding how metabolism coordinates immune cell function might uncover innovative therapeutics or metabolic adjuncts to reorient cells toward immune protection (Cheng et al., 2016).

The adaptation of the fungus to its host also requires a profound reprogramming of the fungal transcriptome. Previous studies have been centered on the isolation of minute amounts of RNA from host tissues and the use of microarrays or RNA-sequencing (Cairns et al., 2010; Liu et al., 2015). Amorim-Vaz and Sanglard discuss two emerging technologies to improve the capture of fungal RNA and discuss their pros and cons, and how microbial transcriptomics can benefit from them (Amorim-Vaz and Sanglard). The underrepresentation of fungal DNA has also been hampering the precise characterization of the fungal communities in the human host, their composition and dynamics, and contribution to disease. By examining the human gut mycobiota, Strati et al. highlight important implications of age- and gender-dependent interindividual variation in microbiota diversity, and consequently susceptibility to fungal infection (Strati et al.).

Systems biology has demonstrated that the fungal cell wall is a highly dynamic organelle (Brown et al., 2015). Based on the proteomics of fungal extracellular vesicles (EVs), Nimrichter et al. discuss the contributions of EVs to the interaction with host cells (Nimrichter et al.). Likewise, fungal sphingolipids form a unique and complex group of bioactive lipids with a role in microbial pathogenesis (Bryan et al., 2015). Singh and Del Poeta provide an overview of the methods employed in qualitative and quantitative fungal sphingolipidomics (Singh and Del Poeta). The characterization of the cell wall composition and dynamics is therefore expected to deliver novel therapeutic and vaccination targets (Carvalho et al., 2012a). On the other hand, Prado et al. demonstrate that *Paracoccidioides lutzii* undergoes a global metabolic adaptation in response to the antifungal argentilactone (Prado et al.). Thus, the use of omics may extend beyond therapeutic target identification to the evaluation of the course of action of antifungals and mechanisms of resistance.

Among fungi, the biology of *C. albicans* is unique due to the flexible reassignment of the leucine CUG codon to serine and synthesis of statistical proteins (Gomes et al., 2007). This aminoacid misincorporation shapes cell surface composition (Miranda et al., 2013) and drives vast phenotypic diversity concerning metabolism, drug resistance and host immunity (Bezerra et al., 2013). Simões et al. resequenced the genome of mistranslating strains to infer that expression of serine tRNAs was linked to mutations in the deneddylase gene (Simões et al.). Besides demonstrating neddylation as a key mechanism in the tolerance to codon ambiguity, this posttranslational modification is highlighted as a promising therapeutic target.

In conclusion, the articles presented here provide an overview of the potential for omics based on concrete examples of their application. With the unrelenting advances in technology, a major contribution of omics to the elucidation of fungal pathogenesis are anticipated, providing crucial information bridging basic research to the patient's bedside.

## Author contributions

AC and GG conceived and designed the work, drafted the article, and provided final approval of the version to be published.

## Funding

This work was supported by the Northern Portugal Regional Operational Programme (NORTE 2020), under the Portugal 2020 Partnership Agreement, through the European Regional Development Fund (FEDER) (NORTE-01-0145-FEDER-000013), Fundação para a Ciência e Tecnologia (FCT) (IF/00735/2014 to AC), Conselho Nacional de Desenvolvimento Científico e Tecnológico (CNPq), Brazil, and Fundação de Amparo a Pesquisa do Estado de São Paulo (FAPESP), Brazil.

### Conflict of interest statement

The authors declare that the research was conducted in the absence of any commercial or financial relationships that could be construed as a potential conflict of interest.
